# Functional pleiotropism, diversity, and redundancy of *Salvia miltiorrhiza* Bunge JAZ family proteins in jasmonate-induced tanshinone and phenolic acid biosynthesis

**DOI:** 10.1093/hr/uhac166

**Published:** 2022-07-25

**Authors:** Pengda Ma, Tianlin Pei, Bingbing Lv, Mei Wang, Juane Dong, Zongsuo Liang

**Affiliations:** College of Life Sciences, Northwest A&F University, Yangling, China; College of Life Sciences, Northwest A&F University, Yangling, China; Shanghai Key Laboratory of Plant Functional Genomics and Resources; College of Life Sciences, Northwest A&F University, Yangling, China; College of Life Sciences, Northwest A&F University, Yangling, China; College of Life Sciences, Northwest A&F University, Yangling, China; College of Life Sciences, Northwest A&F University, Yangling, China; College of Life Sciences and Medicine, Zhejiang Sci-Tech University, Hangzhou, China

## Abstract

Jasmonate (JA) signaling regulates plant growth and development, biotic and abiotic stress tolerance, and primary and secondary metabolism biosynthesis. It is extensively modulated by JA-ZIM-domain (*JAZ*) family genes. In previous work, we obtained nine *SmJAZ* genes of *Salvia miltiorrhiza* and proved that SmJAZ8 was the core repressor of JA-induced tanshinone and phenolic acid biosynthesis. Here, we demonstrate that *SmJAZ3* and *SmJAZ4* act as repressors of JA-induced biosynthesis of tanshinones and salvianolic acid B (Sal B). This suggests that *SmJAZ3/4* are functionally redundant in tanshinone and Sal B biosynthesis. *SmJAZ1/2/5/6/9* are activators of JA-induced tanshinone biosynthesis and repressors of JA-induced Sal B biosynthesis. This demonstrates the redundancy and diversity of *SmJAZ1/2/5/6/9* functions. Besides, *SmJAZ10* inhibited JA-induced Sal B synthesis, but had no effect on the synthesis of tanshinone. Two-hybrid screening (Y2H) showed that SmJAZs formed homologous or heterogeneous dimers. Y2H and firefly luciferase complementation imaging (LCI) assays revealed that SmJAZs also formed a complex regulatory network with SmMYC2a, SmMYC2b, SmMYB39, and SmPAP1. Quantitative reverse transcription-PCR (qRT-PCR) indicated that *SmJAZs* regulated each other at the transcriptional level. Herein, we prove that *SmJAZ*s have functional pleiotropism, diversity, and redundancy in JA-induced tanshinone and phenolic acid biosynthesis. This study provides an important clue for further understanding the inherent biological significance and molecular mechanisms of the *JAZ* family as the gene number increases during plant evolution.

## Introduction

*Salvia miltiorrhiza* Bunge is a perennial herb. Its dried roots and rhizomes are among the core traditional medicinal herbs in China and have been widely used in the prevention and treatment of coronary heart disease, chronic renal failure, arteriosclerosis, and other diseases. The active components of *S. miltiorrhiza* are mainly lipid-soluble tanshinones and water-soluble phenolic acids. These include tanshinone IIA (TA IIA), cryptotanshinone (CT), tanshinone I (TA I), dihydrotanshinone I (DT I), salvianolic acid A (Sal A), salvianolic acid B (Sal B), rosmarinic acid (RA), protocatechualdehyde and so on [1, 2]. Among the rosin-type diterpenoids, tanninones are derived from isopentenyl diphosphate (IPP), the result of the reaction between mevalonic acid (MVA) and 2,4-d-erythritol-4-phosphate (MEP). The phenylpropanoid pathway, which also produces tyrosine-derived phenolic acids, and the tyrosine-derived pathway are both involved in the production of phenolic acids. By combining 4-coumaroyl-CoA and 4-hydroxyphenyllactate, rosmarinic acid synthase (RAS) creates 4-coumaroyl-3,4-dihydroxyphenyllactic acid (4C-DHPL). Finally, CYP98A14 catalyzes the transformation of 4C-DHPL to RA. However, the pathway for subsequent synthesis of salvianolic acid has not been completely resolved [3].

Jasmonates (JAs) act as phytohormones in land plants to regulate growth and development, biotic and abiotic stress tolerance, and primary and secondary metabolism biosynthesis [4]. JAZ family repressors recruit the NINJA/TPL co-repressors and interact with downstream transcription factors to repress JA responses. The JA receptor Coronatine Insensitive 1 (COI1) destroys bioactive JAs by targeting the JAZ molecule and causing downstream transcription factors to be released [5]. In *S. miltiorrhiza*, methyl jasmonate (MeJA) promotes the expression of transcription factors, including v-myb myeloblastosis viral oncogene homolog (MYB), basic helix–loop–helix (bHLH), ethylene response factor (ERF), WRKY, and lateral organ boundaries domain (LBD), which are involved in the positive or negative regulation of tanshinone and phenolic acid synthesis. For example, *SmMYB9b* [6], *SmMYB97* [7], *SmMYC2a* [8], and *SmERF73* [9] are the positive regulators of tanshinone synthesis, while SmERF115 [10] is a negative regulator. Besides, *SmMYB1* [11], *SmMYB97* [7], and *SmPAP1* [12] are positive regulators of tannic acid synthesis, while *SmbHLH37* [13], *SmERF1LI* [14], and *SmLBD50* [15] are negative regulators. These transcription factors are repressed by the JAZ protein, which in turn inhibits the different JA responses.

With the continuous evolution of terrestrial plants, the number of JAZ family members is increasing. The number of *JAZ* genes ranges from 1 in the liverwort *Marchantia polymorpha* to 50 in wheat (*Triticum aestivum*) but most species contain 10–20 [16–21]. The single *M. polymorpha MpJAZ* gene may regulate more than one biological function, including senescence, plant defenses, repressing jasmonate biosynthesis, and promoting cell growth and reproductive fitness [22]. With the continuous amplification and increasing number of *JAZ* genes in terrestrial plants, the *JAZ* family genes retain this pleiotropism and also offer diversity and redundancy. Diversity allows JAZ family members to regulate different biological functions, while redundancy appears when multiple JAZ family members regulate a given biological function. For example, overexpression (full-length or ΔJas) or knockout [T-DNA insertion mutants and RNA interference (RNAi)] studies on single *Arabidopsis JAZ* genes indicate that each *Arabidopsis JAZ* family gene except *JAZ5* regulates one or more biological functions [23–26]. *AtJAZ9* and *AtJAZ11* regulate flowering [27], *AtJAZ12* affects root growth [28], and *AtJAZ13* regulates insect resistance [29]. These four *JAZ* genes regulate single but different types of biological function, which indicates the diversity of *JAZ* genes in *Arabidopsis*. Other *Arabidopsis JAZ* genes regulate two to eight biological functions, with *AtJAZ1/4*/*7* each regulating eight biological functions. *AtJAZ1* regulates root growth, hypocotyl growth, flowering, male sterility, insect resistance, bacterial pathogen resistance, freezing tolerance, and drought tolerance [30–32]. *AtJAZ4* regulates root growth, hypocotyl growth, root hair development, flowering, bacterial pathogen resistance, leaf senescence, freezing tolerance, and anthocyanin accumulation [27, 33–35]. *AtJAZ7* regulates root growth, shoot secondary growth, flowering, leaf senescence, fungal resistance, bacterial pathogen resistance, drought tolerance, and primary and specialized metabolism [36–38]. Thus individual *JAZ* genes exhibit pleiotropism. At the same time, these biological functions are regulated by multiple (two to nine) *Arabidopsis JAZ* genes. Hypocotyl growth [35], shoot secondary growth [36], root hair development [39], male sterility [30], freezing tolerance [34], drought tolerance [38], and primary and specialized metabolism [37] are regulated by two *Arabidopsis JAZ* genes, while root growth is regulated by up to eight *JAZ* genes, including *AtJAZ1/2/3/4/7/8/10/12* [23, 24, 30, 40, 41]. In addition, although there are no reports of an independent function of *AtJAZ5*, analysis of double mutants of *AtJAZ5* and *10* shows that they cooperate to regulate bacterial pathogen resistance [42]. These results all demonstrate that *JAZ* genes have diversity and redundancy. However, whether JAZ in *S. miltiorrhiza* is also diverse and redundant in the synthesis of salvianolic acid and tanshinone is unclear.

Studies on JAZ proteins in *S. miltiorrhiza* have been reported. Overexpression and RNAi technology have revealed that SmJAZ3 and SmJAZ9 are involved in regulating the synthesis of tanshinones in *S. miltiorrhiza* hairy roots [43]. Sal B content was increased in *Salvia* transgenic seedlings overexpressing SmJAZ8 [44]. Yeast two-hybrid (Y2H) analysis showed that SmJAZ1 and SmJAZ2 interacted with the tanshinolate and tanshinone synthesis regulators SmMYC2a and SmMYC2b, respectively [45]. A previous study analyzed differentially expressed genes in *S. miltiorrhiza* roots using cDNA–amplified fragment length polymorphism (AFLP) and found that one of the *JAZ* genes was upregulated by yeast extract action, and yeast extract inducers could significantly promote the accumulation of tanshinones [46]. Overexpression and RNAi in hairy roots revealed SmJAZ8 to be a key negative regulator of JA-induced tanshinone and phenolic acid biosynthesis [47]. The specific function of other *SmJAZ*s in JA-induced biosynthesis of tanshinones and phenolic acids was unclear. In this study, we constructed hairy roots of *S. miltiorrhiza* with overexpression of *SmJAZ*s, and confirmed the functional pleiotropism, diversity, and redundancy of *SmJAZ*s in JA-induced tanshinone and phenolic acid biosynthesis by detection of target metabolites and key enzyme genes in the biosynthesis pathways. *SmJAZ3* has the same function as *SmJAZ4*, while *SmJAZ1*/*2*/*5*/*6*/*9* play the opposite role in the JA-induced biosynthesis of tanshinones and Sal B. *SmJAZ10* is the only repressor of Sal B synthesis. This study further clarifies the specific mechanisms of *SmJAZ*s in regulating the two metabolic pathways at the same time and proves that *SmJAZ*s regulate each other at the transcriptional and protein levels, while SmJAZs and multiple transcription factors form a complex regulatory network.

## Results

### Identification and characterization of *SmJAZ* gene family members

**Figure 1:**
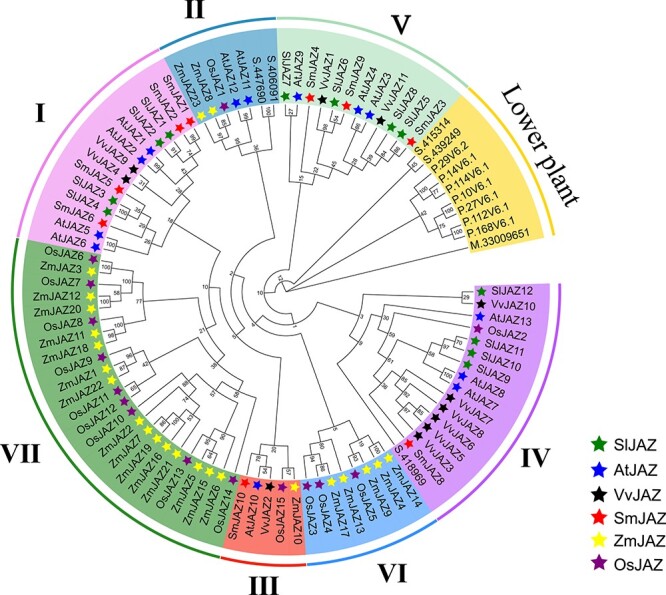
Phylogenetic tree of JAZs from different species. Sm, *Salvia miltiorrhiza*; At, *Arabidopsis thaliana*; Zm, *Zea may*; Os, *Oryza sativa*; Vv, *Vitis vinifera*; Sl, *Solanum lycopersicum*; Pp, *Physcomitrella patens*; Smo, *Selaginella moellendorffii*; Mp, *Marchantia polymorpha.* S. 418969, *S. moellendorffii_418 969*; S. 447690, *S. moellendorffii_447 690*; S. 406091, *S. moellendorffii_406 091*; S. 415314, *S. moellendorffii_415 314*; S. 439249, *S. moellendorffii_439 249*; P. 29 V6.2, *P. patens_Pp1s103_29V6.2*; P. 14 V6.1, *P. patens_Pp1s442_14V6.1*; P. 114 V6.1, *P. patens_Pp1s88_114V6.1*; P. 10 V6.1, *P. patens_Pp1s442_10V6.1*; P. 27 V6.1, *P. patens_Pp1s103_27V6.1*; P. 112 V6.1, *P. patens_Pp1s88_112V6.1*; P. 168 V6.1, *P. patens_Pp1s15_168V6.1*; M. 33 009 651, *M. polymorpha_33 009 651*.

To predict the evolution of the expanded *JAZ* members in *S. miltiorrhiza*, we assembled for phylogenetic analysis full-length *JAZ*s from *Arabidopsis thaliana* (13 *AtJAZ*s), *Oryza sativa* (15 *OsJAZ*s), *Vitis vinifera* (12 *VvJAZ*s), and *S. miltiorrhiza* (9 *SmJAZ*s), plus *Selaginella moellendorffii* (5 *SemJAZ*s), *Physcomitrella patens* (6 *PpJAZ*s), and *M. polymorpha* (1 *MpJAZ*), which represent the earlier branching land plant lineage. The single identified *MpJAZ* was used as the outgroup. This phylogenetic analysis allowed us to classify the *JAZ*s from higher plants into seven main groups (groups I–VII) (Fig. 1). Groups I and V contained only *JAZ*s from dicots, while groups VI and VII included *JAZ*s only from monocots. As seen from the branch structure of the phylogenetic tree, the *JAZ*s in group V seem to have appeared earliest, followed by groups IV, VI, II, and III, and the last to arise were groups I and VII. *SmJAZ*s were clustered into group I (*SmJAZ1/2/5/6*), group III (*SmJAZ10*), group IV (*SmJAZ8*), and group V (*SmJAZ3/4/9*). The exon–intron structure of the *SmJAZ*s is variable. The number of exons present varied from three to seven, with *SmJAZ1/2/8* having three exons, *SmJAZ5/6/10* having five exons, *SmJAZ4* having six exons, and *SmJAZ3* and *SmJAZ9* having seven exons each (Supplementary Data Fig. S1). Conserved domain analyses found that all SmJAZs contained ZIM and Jas domains (Supplementary Data Fig. S1), while as we reported previously SmJAZ8 possessed a non-canonical Jas domain lacking a short conserved motif [47]. In addition, SmJAZ1/2/5/6/8 had LxLxL-type EAR motifs and SmJAZ10 possessed a CMID domain, each located at their N termini.

### Expression patterns of SmJAZs

**Figure 2:**
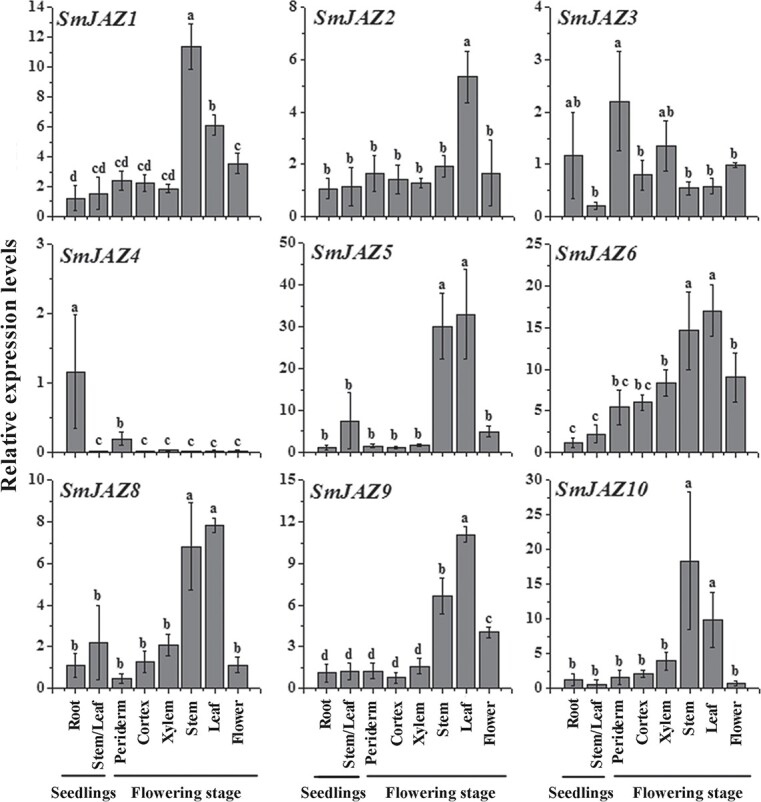
Tissue-specific expression of *SmJAZs.* Bars are mean ± standard deviation from three independent biological replicates. Different letters indicate significant differences among means (*P* < .05). Expression levels of genes in roots of seedlings were set to 1.

The expression levels of SmJAZs in different tissues of *S. miltiorrhiza* seedlings and over the flowering period were determined utilizing quantitative reverse transcription–PCR (qRT–PCR). Except for *SmJAZ3* and *SmJAZ4*, the expression levels of *SmJAZs* at the flowering stage were generally higher than that at the seedling stage (Fig. 2). During the seedling stage, the expression level of *SmJAZ4* in roots was significantly higher than those in stems and leaves, while the expression of other *SmJAZs* showed no difference. Besides, *SmJAZs* exhibited tissue specificity of expression during the flowering stage. The expression of *SmJAZ3* and *SmJAZ4* in roots was higher than in the aerial part, and *SmJAZ4* was specifically expressed in the root periderm. The expression levels of other *SmJAZs* in the aerial part were higher than those in the roots. *SmJAZ1*/*2*/*9* had the highest expression in stems, while *SmJAZ5*/*6*/*8*/*10* were expressed highly in stems and leaves.

The Pearson’s correlation coefficients (*r*) between gene expression profiles of SmJAZs, phenolic acids, and tanshinones were calculated. The expression of *SmJAZ1* was markedly negatively correlated with the accumulation of Sal B (which had the highest accumulation in periderm and cortex) and total phenolic acids (TPAs), which could be detected in all tissues (Supplementary Data Table S4 and Supplementary Data Fig. S2). The expressions of *SmJAZ3* and *SmJAZ4* were significantly positively correlated with concentrations of tanshinones, which were specifically accumulated in roots (Supplementary Data Table S4 and Supplementary Data Fig. S2). Besides, *SmJAZ6* expression was markedly negatively correlated with tanshinone accumulation, while the expression of *SmJAZ8* had a marked negative correlation with TA IIA accumulation, and the expression of *SmJAZ10* was markedly negatively correlated with TPA accumulation (Supplementary Data Table S4 and Supplementary Data Fig. S2).

### Analysis of interaction between *SmJAZ*s by yeast two-hybrid assay

**Figure 3:**
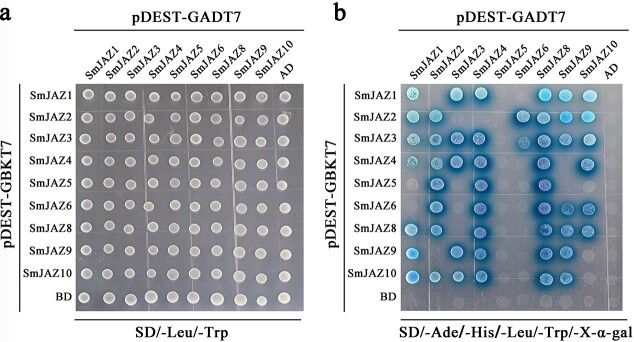
Analysis of interaction between SmJAZs by Y2H assay. **a** Yeast cells grew in SD/−Leu/−Trp plates after transformation; **b** Yeast cells grew in SD/−Ade/–His/−Leu/−Trp/+X-α-Gal plates after transformation.

To investigate the mutualistic relationship between SmJAZ proteins, a yeast two-hybrid experiment was conducted. The white plaques could be grown on all transformants on SD/−Leu/−Trp plates, indicating that plasmid transformation was successful (Fig. 3a). The negative controls (pDEST-GBKT7 + pDEST-GADT7-SmJAZ, pDEST-GBKT7-SmJAZ+pDEST-GADT7, and pDEST-GBKT7 + pDEST-GADT7) could not grow normally on SD/−Ade/−His/−Leu/−Trp/+X-α-gal plates (Fig. 3b). From the growth of blue yeast plaques on plates deficient in four amino acids, except for SmJAZ5 and SmJAZ6, all SmJAZs can interact with themselves, indicating that they can form homodimers (Fig. 3b). In addition, most SmJAZs can interact with other SmJAZs, indicating that heterodimers can be formed. When SmJAZ5 was connected to pDEST-GADT7 vector, its interaction with other SmJAZs could not be detected, but when SmJAZ5 was connected to pDEST-GBKT7 vector, it could interact with SmJAZ2/4/8 (Fig. 3b). When SmJAZ8 was connected to pDEST-GADT7 vector, SmJAZ8 could interact with all other SmJAZs, but when SmJAZ8 was connected to pDEST-GBKT7 vector, it could interact only with a few SmJAZs.

### Distinct roles of *SmJAZ*s in jasmonate-induced biosynthesis of salvianolic acids and tanshinones

To further investigate whether the various *SmJAZ* genes exhibit divergent functions in regulating JA-induced biosynthesis of phenolic acids and tanshinones, transgenic hairy roots overexpressing the full-length open reading frame (ORF) of each *SmJAZ*, as well as the empty vector control (EV), were prepared separately. The positive transgenic hairy roots were identified by the presence of red fluorescent protein (data not shown) and by gene-specific primer amplification of the CaMV 35S promoter and partial *SmJAZ* gene (Supplementary Data Fig. S3). The SmJAZ transcripts in the corresponding transgenic lines were significantly upregulated compared with their control counterparts, as determined by qRT–PCR (Supplementary Data Fig. S4). HPLC was used to detect the MeJA-induced accumulation of RA, Sal B, and four tanshinones (TA IIA, CT, TA I, and DT I) in *SmJAZ*-overexpressing transgenic hairy roots. There was no significant difference between the contents of phenolic acids and tanshinones in empty vector control 6 (EV6) and empty *Agrobacterium rhizogenes* ATCC15834 control 3 (ATCC3) (Tables 1 and 2). However, overexpression of *SmJAZs* significantly increased MeJA-induced RA accumulation in transgenic hairy roots. The transgenic lines with the highest and lowest accumulation were *SmJAZ2*-overexpressing line 2 (J2O2) [73.10 ± 3.24 mg/g dry weight (DW)] and *SmJAZ6*-overexpressing line 6 (J6O6) (10.31 ± 0.5 mg/g DW), reaching respectively 9.5 and 1.35 times that present in ATCC3 (7.66 ± 1.97 mg/g DW) (Table 1). Overexpression of all *SmJAZs* significantly inhibited MeJA-induced accumulation of Sal B in transgenic hairy roots (Table 1). The lines with the highest and lowest contents were J5O3 (50.68 ± 3.27 mg/g DW) and J2O6 (15.45 ± 2.02 mg/g DW); these contents are 16 and 74% lower than that in the control ATCC3 (60.19 ± 5.71 mg/g DW) (Table 1). The accumulation of TPA did not change significantly in *SmJAZ5-* and *SmJAZ9*-overexpressing hairy roots, but the TPA accumulation of line J2O2 increased significantly under MeJA treatment, and the TPA accumulation of other *SmJAZ*-overexpressing hairy root lines decreased significantly compared with ATCC3 under MeJA treatment (Table 1).

**Table 1:** Analysis of salvianolic acid production from *SmJAZ*-overexpressing hairy root lines under MeJA treatment.

Vector		Compound		
	Line	RA mg/g DW	Sal B mg/g DW	TPA mg/g DW
ATCC15834	ATCC3	7.66 ± 1.97	60.19 ± 7.30	67.86 ± 5.87
pK7WG2R-EV	EV6	6.50 ± 0.44	58.65 ± 0.97	65.15 ± 0.54
pK7WG2R-*SmJAZ1*	J1O1	20.75 ± 2.26^**^	43.47 ± 1.92^**^	64.22 ± 1.80
	J1O20	16.81 ± 2.89^**^	35.37 ± 3.61^**^	52.18 ± 5.94^*^
pK7WG2R-*SmJAZ2*	J2O2	73.10 ± 3.24^**^	16.79 ± 0.53^**^	89.89 ± 3.78^*^
	J2O6	64.15 ± 9.49^**^	15.45 ± 2.02^**^	79.60 ± 11.51
pK7WG2R-*SmJAZ3*	J3O3	16.62 ± 1.36^**^	26.10 ± 1.81^**^	42.71 ± 3.17^**^
	J3O5	16.50 ± 0.07^**^	33.57 ± 5.79^**^	50.08 ± 5.74^*^
pK7WG2R-*SmJAZ4*	J4O4	14.52 ± 1.30^*^	17.32 ± 0.54^**^	31.85 ± 1.83^**^
	J4O10	16.88 ± 2.40^**^	23.12 ± 0.64^**^	40.00 ± 3.04^**^
pK7WG2R-*SmJAZ5*	J5O1	30.00 ± 4.78^**^	42.28 ± 6.24^*^	72.28 ± 11.03
	J5O3	26.28 ± 2.15^**^	50.68 ± 3.27^*^	76.96 ± 5.42
pK7WG2R-*SmJAZ6*	J6O4	10.83 ± 2.32	19.80 ± 5.06^**^	30.63 ± 7.38^**^
	J6O6	10.31 ± 0.5^*^	18.71 ± 3.54^**^	29.01 ± 4.03^**^
pK7WG2R-*SmJAZ9*	J9O4	60.77 ± 5.14^**^	17.88 ± 1.53^**^	78.64 ± 6.67
	J9O14	56.61 ± 1.65^**^	17.55 ± 1.06^**^	74.16 ± 2.71
pK7WG2R-*SmJAZ10*	J10O7	16.49 ± 1.12^**^	22.26 ± 1.35^**^	38.74 ± 2.47^**^
	J10O10	19.65 ± 0.15^**^	20.87 ± 1.12^**^	40.52 ± 1.27^**^

Statistical analyses were performed by Student’s *t*-test, and three independent biological replicates were set for each line. Asterisks in the graph indicate significant differences between each transgenic strain and control ATCC3 at the level of .01 < *P* < .05 (^*^) or *P* < .01 (^**^).

**Table 2:** Analysis of tanshinone production from *SmJAZ*-overexpressing hairy root lines under MeJA treatment.

Vector	Compound					
	Line	DT I mg/g DW	CT mg/g DW	TA I mg/g DW	TA IIA mg/g DW	TTA mg/g DW
ATCC15834	ATCC3	0.39 ± 0.02	0.27 ± 0.03	0.69 ± 0.11	0.36 ± 0.02	1.71 ± 0.12
pK7WG2R-EV	EV6	0.38 ± 0.01	0.27 ± 0.02	0.64 ± 0.31	0.33 ± 0.02	1.62 ± 0.36
pK7WG2R-*SmJAZ1*	J1O1	0.35 ± 0.05	0.28 ± 0.06	1.18 ± 0.46	0.33 ± 0.04	2.14 ± 0.46
	J1O20	0.47 ± 0.13	0.24 ± 0.09	1.22 ± 0.17^**^	0.32 ± 0.06	2.25 ± 0.43
pK7WG2R-*SmJAZ2*	J2O2	0.54 ± 0.11	0.36 ± 0.08	1.63 ± 0.46^*^	0.34 ± 0.01	2.87 ± 0.65^*^
	J2O6	0.62 ± 0.08^*^	0.5 ± 0.23	1.33 ± 0.19^*^	0.38 ± 0.04	2.84 ± 0.43^*^
pK7WG2R-*SmJAZ3*	J3O3	0.32 ± 0.05	0.15 ± 0^*^	0.77 ± 0.18	0.34 ± 0.02	1.58 ± 0.2
	J3O5	0.33 ± 0.07	0.17 ± 0.03^*^	0.98 ± 0.37	0.36 ± 0.04	1.83 ± 0.5
pK7WG2R-*SmJAZ4*	J4O4			0.7 ± 0.21	0.36 ± 0.02	1.06 ± 0.22^*^
	J4O10			0.29 ± 0.41	0.19 ± 0.26	0.48 ± 0.67^*^
pK7WG2R-*SmJAZ5*	J5O1	0.87 ± 0.07^**^	0.52 ± 0.03^**^	1.17 ± 0.13^*^	0.36 ± 0.01	2.92 ± 0.22^**^
	J5O3	0.5 ± 0.04^*^	0.28 ± 0.02	1.29 ± 0.01^**^	0.38 ± 0.02	2.45 ± 0.09^**^
pK7WG2R-*SmJAZ6*	J6O4	0.46 ± 0.22	0.31 ± 0.06	1.23 ± 0.33^*^	0.42 ± 0.04	2.42 ± 0.57^*^
	J6O6	0.51 ± 0.11	0.3 ± 0.02	1.36 ± 0.06^**^	0.4 ± 0.07	2.57 ± 0.12^**^
pK7WG2R-*SmJAZ9*	J9O4	0.7 ± 0.12^*^	0.48 ± 0.03^**^	1.16 ± 0.23^*^	0.33 ± 0.02	2.67 ± 0.31^**^
	J9O14	0.96 ± 0.21^*^	0.84 ± 0.01^**^	1.81 ± 0.61^*^	0.58 ± 0.01^**^	4.19 ± 0.8^**^
pK7WG2R-*SmJAZ10*	J10O7	0.37 ± 0.02	0.28 ± 0.01	0.73 ± 0.02	0.33 ± 0.05	1.71 ± 0.1
	J10O10	0.37 ± 0.04	0.23 ± 0.04	0.86 ± 0.03	0.34 ± 0.05	1.8 ± 0.15

The statistical analyses were performed by Student’s *t*-test, and three independent biological replicates were set for each line. Asterisks in the graph indicate significant differences between each transgenic strain and control ATCC3 at the level of .01 < *P* < .05 (^*^) or *P* < .01 (^**^).

Overexpression of *SmJAZs* significantly affected the MeJA-induced accumulation of tanshinones in hairy roots, but different genes produced different effects (Table 2). The accumulation of DT I in each of transgenic line J2O6 with overexpression of *SmJAZ2*, lines J5O1 and J5O3 with overexpression of *SmJAZ5*, and J9O4 and J9O14 with overexpression of *SmJAZ9* was significantly higher than that of ATCC3 under MeJA treatment, and the line with the highest levels, J9O14 (0.96 ± 0.21 mg/g DW), contained 2.5 times the concentration found in ATCC3 (0.39 ± 0.02 mg/g DW). The accumulation of CT in transgenic line J5O1 with overexpression of *SmJAZ5* and transgenic lines J9O4 and J9O14 with overexpression of *SmJAZ9* were significantly higher than that of ATCC3 under MeJA treatment, and the line with the highest concentration, J9O14 (0.84 ± 0.01 mg/g DW), contained levels 3.1 times higher than that of ATCC3 (0.27 ± 0.03 mg/g DW). Overexpression of *SmJAZ3* significantly reduced the accumulation of CT in hairy roots induced by MeJA. DT I and CT were not detected in transgenic hairy root lines overexpressing *SmJAZ4*. The accumulation of TA I in transgenic line J1O20 with overexpression of *SmJAZ1*, lines J2O2 and J2O6 with overexpression of *SmJAZ2*, J5O1 and J5O3 with overexpression of *SmJAZ5*, J6O4 and J6O6 with overexpression of *SmJAZ6*, and J9O4 and J9O14 with overexpression of *SmJAZ9* was significantly higher than that of ATCC3 under MeJA treatment. The strain with the highest concentration, J9O14 (1.81 ± 0.61 mg/g DW), had levels 2.6 times greater than that of ATCC3 (0.69 ± 0.11 mg/g DW). Only the accumulation of TA IIA in transgenic line J9O14 with overexpression of *SmJAZ9* (0.58 ± 0.01 mg/g DW) was significantly higher than that in ATCC3 (0.36 ± 0.02 mg DW) under MeJA treatment. The content of total tanshinones (TTA, the sum of DT I, CT, TA I, and TA IIA) in MeJA-induced transgenic hairy roots overexpressing *SmJAZ2*/*5*/*6*/*9* was significantly higher than that in ATCC3, while the accumulation of TTA in MeJA-induced transgenic hairy roots overexpressing *SmJAZ4* was significantly lower than that in ATCC3. It was also found that the accumulation of tanshinones in transgenic hairy roots overexpressing *SmJAZ10* showed no significant change compared with the control under MeJA treatment. To sum up, under the induction of MeJA overexpression of *SmJAZs* markedly reduced Sal B accumulation in hairy roots and overexpression of *SmJAZ1*/*2*/*5*/*6*/*9* significantly increased tanshinone accumulation in hairy roots, while overexpression of *SmJAZ3* and *SmJAZ4* decreased tanshinone accumulation in hairy roots.

### Expression of genes related to phenolic acid and tanshinone biosynthesis in *SmJAZ* transgenic hairy roots induced by methyl jasmonate

**Figure 4:**
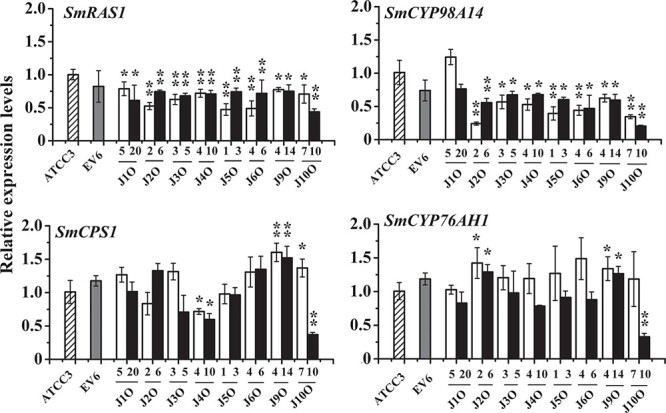
Expression levels of related genes involved in salvianolic acid and tanshinone biosynthesis in transgenic hairy root lines with MeJA treatment. Bars are means ± standard deviation from three independent biological replicates. One asterisk (^*^) indicates a significant difference (.01 < *P* < .05) and two asterisks (^**^) indicate a very significant difference (*P* < .01) between the control and transgenic hairy root lines. Expression levels of the respective genes in ATCC line were set to 1.

The expressions of the phenolic acid-related synthase genes *SmRAS1* and *SmCYP98A14* and the tanshinone-related synthase genes *SmCPS1* and *SmCYP76AH1* in transgenic hairy roots were determined by qRT–PCR. There was no significant difference in the expression of related synthase genes between controls EV6 and ATCC3 (Fig. 4). Overexpression of *SmJAZ*s markedly decreased the expression of *SmRAS1* in transgenic hairy roots induced by MeJA, while, except for *SmJAZ1*, overexpression of other *SmJAZ*s significantly decreased the expression of *SmCYP98A14* in transgenic hairy roots induced by MeJA (Fig. 4).

After MeJA treatment, the expression of *SmCPS1* was significantly decreased in overexpressing *SmJAZ4* transgenic lines J4O4 and J4O10 and overexpressing *SmJAZ10* transgenic line J10O10, and significantly increased in *SmJAZ9* transgenic lines J9O4 and J9O14 and overexpressing *SmJAZ10* transgenic line J10O7, but there was no change in other transgenic lines (Fig. 4). After MeJA treatment, *SmCYP76AH1* expression was markedly increased in transgenic lines J2O2 and J2O6 overexpressing *SmJAZ2* and transgenic lines J9O4 and J9O14 overexpressing *SmJAZ9*, and significantly decreased in transgenic line J10O10 overexpressing *SmJAZ10*, but there was no significant change in other transgenic lines (Fig. 4).

### Expression of other *SmJAZ* in *SmJAZ* transgenic hairy roots induced by methyl jasmonate

**Figure 5:**
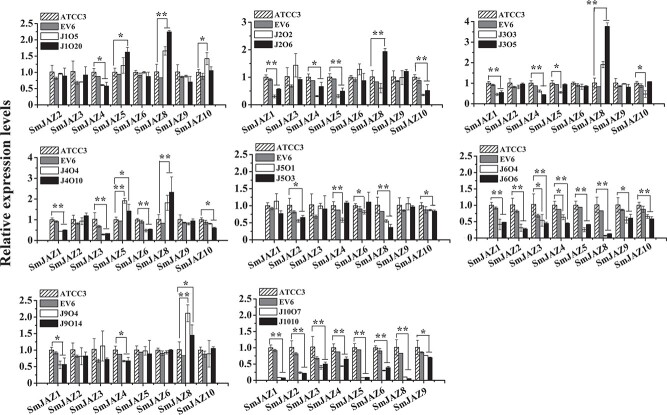
Expression levels of genes of the *SmJAZ* family in transgenic lines with MeJA treatment. Bars are means ± standard deviation from three independent biological replicates. One asterisk (^*^) indicates a significant difference (.01 < *P* < .05) and two asterisks (^**^) indicate a very significant difference (*P* < .01) between the control and transgenic lines. Expression levels of genes in the ATCC line were set to 1.

In order to study the co-regulation between *SmJAZ*s, qRT–PCR was utilized to examine the expression of other members of this gene family in *SmJAZ* transgenic hairy roots induced by MeJA. Transgenic lines EV6 and ATCC3 did not significantly differ in their gene expression levels but numerous effects were observed for lines overexpressing individual *SmJAZ* genes (Fig. 5). Overexpression of *SmJAZ1* significantly inhibited the expression of *SmJAZ4* and promoted *SmJAZ5* (in J1O20), *SmJAZ*8 and *SmJAZ*10 (in J1O5) expression (Fig. 5). Overexpression of *SmJAZ2* significantly inhibited the expression of *SmJAZ1*/*4*/*5*/*10*, and promoted *SmJAZ8* expression in J2O6 (Fig. 5). Overexpression of *SmJAZ3* significantly suppressed the expression of *SmJAZ1*/*4*/*5*/*10* in J3O3, and promoted *SmJAZ8* expression (Fig. 5). Overexpression of *SmJAZ4* significantly inhibited the expression of *SmJAZ1*/*3/6*/*10* and significantly promoted the expression of *SmJAZ5* and *SmJAZ8* (Fig. 5). Overexpression of *SmJAZ5* significantly suppressed the expression of *SmJAZ2*/*8*/*10*, as well as *SmJAZ4* and *SmJAZ6* in J5O1 (Fig. 5). Our previous study demonstrated that overexpression of *SmJAZ8* markedly inhibited the expression of *SmJAZ1*, *5*, and *10*, as well as *SmJAZ4* and *6* in J8O1 [47]. Overexpression of *SmJAZ9* significantly inhibited the expression of *SmJAZ1* and *SmJAZ4* and significantly promoted the expression of *SmJAZ8* (Fig. 5). In addition, overexpression of *SmJAZ6* and *SmJAZ10* significantly suppressed the expression of all *SmJAZ*s except for themselves (Fig. 5).

### Analysis by Y2H and firefly luciferase complementation imaging (LCI) assays of interaction between *SmJAZ*s and transcription factors

**Figure 6:**
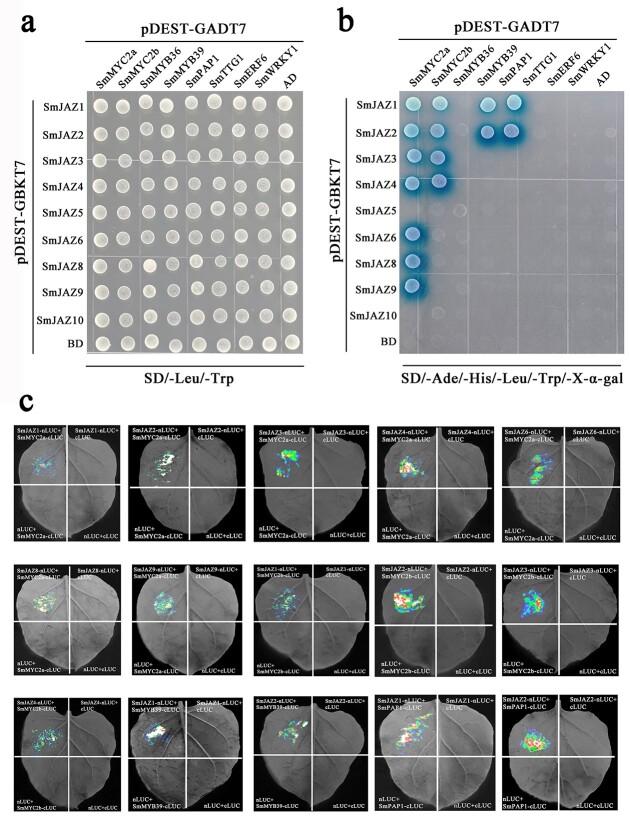
Analysis of interaction between SmJAZs and transcription factors by Y2H and LCI assays. **a** Yeast cells grew in SD/−Leu/−Trp plates after transformation. **b** Yeast cells grew in SD/−Ade/–His/−Leu/−Trp/+X-α-Gal/+AbA plates after transformation. **c** Interaction of SmJAZs and transcription factors in the LCI assays.

White plaques could be grown on all transformants on SD/−Leu/−Trp plates, indicating that plasmid transformation was successful (Fig. 6a). The negative controls (pDEST-GBKT7 + pDEST-GADT7-TFs, pDEST-GBKT7-SmJAZs + pDEST-GADT7 and pDEST-GBKT7 + pDEST-GADT7) could not grow normally on SD/−Ade/-His/−Leu/−Trp/+X-α-Gal/+AbA plates (Fig. 6b). However, the yeast that transformed pDEST-GBKT7-SmJAZ1/2/3/4/6/8/9 and pGADT7-SmMYC2a, pGBKT7-SmJAZ1/2/3/4 and pGADT7-SmMYC2b, pGBKT7-SmJAZ1/2 and pGADT7-SmMYB39, and pGBKT7-SmJAZ1/2 and pGADT7-SmPAP1 grew clear blue plaques.

Then, we further verified the interaction of the interacting proteins in the Y2H assay by LCI assays. The results showed that cotransformation of SmJAZ1/2/3/4/6/8/9-cLUC and SmMYC2a-nLUC, SmJAZ1/2/3/4-cLUC and SmMYC2b-nLUC, SmJAZ1/2-cLUC and SmMYB39-nLUC, and SmJAZ1/2-cLUC and SmPAP1-nLUC could be detected fluorescently in tobacco leaves (Fig. 6c).

### Analysis of tissue specificity and methyl jasmonate-induced expression of transcription factors

**Figure 7:**
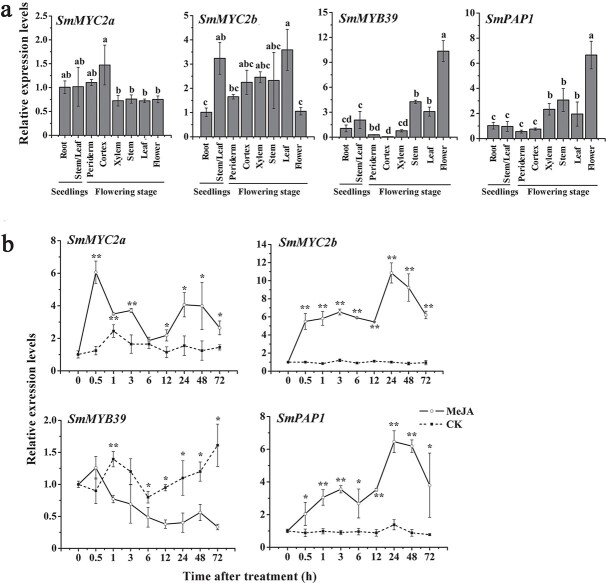
Analysis of expression patterns of transcription factors. **a** Tissue-specific expression of transcription factors. Bars are means ± standard deviation from three independent biological replicates. Different letters indicate significant differences among means (*P* < .05). Expression levels of genes in root from seedlings were set to 1. **b** Transcriptional response of transcription factors to induction by MeJA. Bars are means ± standard deviation from three independent biological replicates. One asterisk (^*^) indicates a significant difference (.01 < *P* < .05) and two asterisks (^**^) indicate a very significant difference (*P* < .01) between control and MeJA-induced hairy roots.

In order to study whether the transcription factors interacting with SmJAZs also have the characteristics of spatiotemporal expression and induced expression, qPCR was employed to examine the expression changes of *SmMYC2a*, *SmMYC2b*, *SmMYB39*, and *SmPAP1* in different tissue parts of *S. miltiorrhiza* at different growth stages and induced by MeJA. In the seedling stage of *S. miltiorrhiza*, *SmMYC2b* expression in stem and leaf were markedly higher than that in root, while the expression of other transcription factors in root, stem, and leaf showed no significant difference (Fig. 7a). In the flowering stage, the expression of *SmMYC2a* in periderm and cortex was the highest, but there was no significant difference in other tissues. The expression of *SmMYC2b* in leaf was significantly higher than that in periderm and flower, but was not significantly different from that in other tissues (Fig. 7a). The expression of *SmMYB39* was the highest in flower, followed by stem and leaf, and the lowest in different root tissues (Fig. 7a). The expression of *SmPAP1* was also the highest in flower, followed by stem, leaf, and xylem, and the lowest in cortex and periderm (Fig. 7a). The change in transcription factor expression under MeJA treatment is shown in Fig. 7b. Expression of *SmMYC2a* increased significantly and reached its highest value after 0.5 hours of treatment, which was 6 times higher than that of the control, and then decreased rapidly, but was still markedly higher than the control (Fig. 7b). *SmMYC2b* and *SmPAP1* expression increased rapidly after treatment and reached the maximum values at 24 h, which were 10.8 and 6.4 times higher than that of the control, respectively (Fig. 7b). After treatment, the expression of *SmMYB39* showed a continuous downward trend compared with the control (Fig. 7b).

## Discussion

### Functional pleiotropism, diversity, and redundancy of *SmJAZ*s

It has been reported that members of the *JAZ* gene family, not only in *Arabidopsis* but also in other plant species, have functional pleiotropism, diversity, and redundancy. In rice, *OsJAZ1* regulates drought and salt tolerance [48, 49], while *OsJAZ8* regulates linalool accumulation and bacterial pathogen resistance [50, 51]. Tobacco *NaJAZd* regulates flower abscission and secondary metabolism, and *NaJAZh* regulates necrosis and/or programmed cell death during plant development and secondary metabolism [52, 53]. *NaJAZ1* and *NaJAZ3* regulate tobacco alkaloid accumulation [54]. Cotton *GhJAZ2* regulates cotton fiber initiation, fungal resistance, insect resistance, and salt tolerance [55–57]. *GhJAZ3* regulates hypocotyl and root growth, trichome formation, and plant height [58]. According to our previous study, SmJAZ8 is a key repressor of JA-induced biosynthesis of phenolic acids and tanshinones [47]. This has shown that SmJAZ8 has functional pleiotropism. This study shows that, apart from *SmJAZ10*, seven other *SmJAZ* genes also regulate Sal B and tanshinones, which proves again the functional pleiotropism of members of the *SmJAZ* gene family.

The functions of the nine *SmJAZ* genes of *S. miltiorrhiza* can be divided into three categories. One is defined by only negative regulation of Sal B, such as by *SmJAZ10*, another is negative regulation of both Sal B and tanshinones, such as by *SmJAZ3*/*4*, and the third is positive and negative regulation of Sal B and tanshinones, such as by *SmJAZ1*/*2*/*5*/*6*/*9*, further indicating the functional pleiotropism and redundancy of members of the *SmJAZ* gene family. Through overexpression and antisense RNA experiments, Shi *et al*. [43] proved that *SmJAZ9* is a negative regulator of tanshinone synthesis, while our experiments show that *SmJAZ9* is a positive regulatory factor. The main reason for this inconsistency is that in our study we primarily investigated the role of *SmJAZ9* induced by MeJA, while Shi *et al*. did not incorporate JA signal induction. RA is considered the biosynthetic precursor of Sal B [59]. We speculate that the increased accumulation of RA is caused by the obstruction of Sal B biosynthesis in SmJAZ-overexpressing lines. In addition to Sal B, there are other derivatives of RA in *S. miltiorrhiza* hairy roots [3], so the TPA content decreases or does not show obvious changes in transgenic hairy roots. In addition, this study and our previous research proved that all nine *SmJAZ* genes are negative regulators of SalB biosynthesis, while *SmJAZ3*/*4*/*8* are negative regulators of tanshinone synthesis [47], and *SmJAZ1*/*2*/*5*/*6*/*9* are positive regulators of tanshinone biosynthesis, indicating that different *SmJAZ*s may have the same or opposite regulation results for the same trait. There are similar results in other species. For example, members of the *JAZ* gene family in *Arabidopsis* showed the same pattern in regulating shoot secondary growth, root hair development, male sterility, freezing tolerance, drought tolerance, anthocyanin accumulation, and other traits. *AtJAZ4*/*7/8* delayed dark-induced leaf senescence, while *AtJAZ6* accelerated leaf senescence [33, 60]. JAZ7 inhibits JA-induced root growth and other *Arabidopsis* JAZs serve as suppressors*.* The above results indicate that all members of the *SmJAZ* protein family of *S. miltiorrhiza* participate in the regulation of JA-induced biosynthesis of tanshinones and phenolic acids acting as repressors and/or activators.

### Mutual regulation of SmJAZs at protein level

This study showed that, except for SmJAZ5 and SmJAZ6, SmJAZs can form homodimers. In addition, most SmJAZs can form heterodimers. The formation of homo- or heterodimers of JAZ has also been reported in other plant species. For example, 7 JAZs (AtJAZ1/2/3/4/5/6/10) can form homodimers, while 11 JAZs (AtJAZ1/2/3/4/5/6/8/9/10/11/12) can form heterodimers, with the ZIM domain mediating the formation of homodimers and heterodimers in *Arabidopsis* [40, 41, 61]. In *Arabidopsis*, AtJAZ3ΔJas, which lacks the Jas domain and loses the ability to bind to transcription factor MYC2, can form heterodimers with intact JAZ proteins, such as JAZ1 and JAZ9, to stabilize the activity of transcription factors and reduce the level of JA signal response [61]. In cotton, GhJAZ2/5/14 can form homodimers. However, 10 JAZs, GhJAZ2/5/7/11/12/14/21/24/26/29, can form heterodimers. The homodimer and heterodimer formed by GhJAZ2-Jas, the truncated form of GhJAZ2, were highly interacting. However, the isolated ZIM domain of GhJAZ2 did not exhibit extensive homodimerization, or heterodimeric interaction with other GhJAZ proteins [56, 57]. Interactions between OsJAZ2/4/5/9/12/15 and OsJAZ8 are observed in rice, but OsJAZ8 does not interact with itself [50].

As the number of JAZs increased with the evolution of plants, they diverged and may be divided into different subgroups according to the characteristics of sequence structure. Clusters found are: group I (*SmJAZ1/2/5/6*), group III (*SmJAZ10*), group IV (*SmJAZ8*), and group V (*SmJAZ3/4/9*). These JAZ subgroups show different tissue expression characteristics. For example, all nine *SmJAZs* show different expression characteristics between the seedling and flowering periods. This study demonstrates that these nine *SmJAZs* have diversity in regulating JA-induced tanshinone and phenolic acid synthesis. However, the nine functional *SmJAZs* will present greater diversity after protein level interactions, because SmJAZs can form homo- or heterodimers, thus allowing them to regulate each other at the protein level, which is one of the forces driving the functional pleiotropism, diversity, and redundancy of *SmJAZ*s.

### Feedback regulation of *SmJAZ*s at the transcriptional level

In this study, overexpression of a specific *SmJAZ* gene significantly promoted or inhibited the expression of other genes. This indicates that *SmJAZs* also regulate each other at the transcriptional level as well as at the protein level. This regulation of JAZs at the transcriptional level has also been reported in other plant species. Since *JAZ*s control the regulation of the downstream target gene through specific transcription factors, the transcriptional level regulation between *JAZs* is also achieved by their interacting transcription factors. This is typical feedback regulation. In *Arabidopsis*, full-length or Jas domain-defective JAZ proteins are regulated by *JAZ*s themselves at transcriptional level through MYCs (MYC2/3/4) or WRKYs (WRKY18 and WRKY40). *JAZ8* is resistant to JA-induced degradation along with mutants lacking a Jas domain, which results in loss of the COI1-interacting degron. The increase in expression of stable JAZ constitutes negative regulatory feedback of the JA signal; thus, the sensitivity to JA is reduced [23, 62, 63]. Rice OsbHLH148 mediates drought resistance by interacting with OsJAZs. Overexpression of *OsbHLH148* in rice can increase the expression levels of *OsJAZ1/2/3/4/5/6/10/11*, suggesting that *OsJAZs* in rice control themselves through upregulated expression at the transcriptional level via *OsbHLH148* [64]. In tobacco, plants with RNAi-induced silencing of *NtJAZ1/3/7a/10* not only have decreased nicotine content but also show regulation of the upregulated or downregulated expression of the other three JAZs, both with and without MeJA treatment. It is suggested that there is mutual regulation of *NtJAZ1/3/7A/10* at the transcriptional level [65].

Besides, *MYC2* acts as an upstream core transcription factor interacting with *JAZs* to initiate downstream *TF* expression, *JAZ*s, and other JA-responsive genes. The G-box is a core *cis*-element of MYC2-targeted JA-responsive genes and plays a key role in initiating downstream gene transcription including that of *JAZs* [23, 66]. *SmMYC2a*, a homologous gene of *Arabidopsis MYC2* that has been shown to interact with *SmJAZ*s in *S. miltiorrhiza*, has a positive role in the regulation of tanshinones and phenolic acids induced by JA, and its downstream target genes all contain G-boxes [47, 67]. We found G-boxes in the promoters of all nine *SmJAZ*s (Supplementary Data Table S6), so this once again provides a basis for the feedback regulation of *JAZ*s at the transcriptional level.

### Interacting regulatory network of SmJAZs-SmTFs

In the JA signaling pathway, the transcription factors interacting with JAZs also show diversity, mainly including bHLH, MYB, WRKY, and other types of transcription factor. The diversity of the interacting transcription factors also lays a foundation for understanding the functional pleiotropism of the JA signaling pathway [68]. This study demonstrated that different SmJAZs interacted with four bHLH and MYB transcription factors, and these four transcription factors showed different tissue and induced expression characteristics. Previous studies have also demonstrated that another five transcription factors, SmMYB97, SmJRB1, SmbHLH37, SmLDB50, and SmERF73, interact with different SmJAZ proteins, and these transcription factors are all involved in the regulation of tanshinones and phenolic acid synthesis [7, 9, 13, 15, 69]. Therefore, SmJAZs and multiple types of transcription factors constitute a complex regulatory network. With further study of *S. miltiorrhiza*, additional transcription factors interacting with SmJAZs may be found to participate in the regulation of tanshinone and phenolic acid metabolic pathways or participate in the regulation of tanshinones and phenolic acids by combining with these transcription factors to form complex upstream and downstream regulatory processes. Thus, the regulation network composed of SmJAZs-SmTFs is complex. This multifaceted regulatory network is another major reason for the functional pleiotropism, diversity, and redundancy of SmJAZs*.*

### Conclusions

In this study, we demonstrated that *SmJAZ3/4* are repressors of JA-induced tanshinone and Sal B biosynthesis. This suggests that SmJAZ3/4 are functionally redundant in tanshinone and Sal B biosynthesis. *SmJAZ1/2/5/6/9* are activators of JA-induced tanshinone biosynthesis and repressors of JA-induced Sal B biosynthesis. This demonstrates the redundancy and diversity of SmJAZ1/2/5/6/9 functions. Besides, SmJAZ10 only inhibited JA-induced Sal B synthesis, but had no effect on the synthesis of tanshinone. SmJAZs form homodimers or heterodimers and form a complex regulatory network with transcription factors. In addition, *SmJAZ*s are mutually regulated at the transcriptional level. Here, we demonstrate that S*mJAZ*s are pleiotropic, diverse, and redundant in JA-induced tanshinone and phenolic acid biosynthesis.

## Materials and methods

### Plant materials and treatments

*S. miltiorrhiza* seeds were purchased from Shaanxi Tianshili Phytopharmaceutical Co. The roots were carefully divided into periderm, xylem, and bast with a surgical blade. The material was baked at 45°C to constant weight and stored in a dry place for composition determination. The *S. miltiorrhiza* hair roots were from the strain kept in our laboratory. We weighed 0.3 g of trichomes and inoculated them aseptically into 250-ml triangular flasks containing 50 ml of 6,7-V liquid medium, and incubated at 25°C, protected from light, in a shaker at 120 r/min^−1^, the subculture period is approximately 30 days.

MeJA was purchased from Shanghai Yuanye Biotechnology Co. (Shanghai, China). Hairy roots of *S. miltiorrhiza* were incubated for 21 days for induction experiments. The samples were divided into two groups, and MeJA and anhydrous ethanol (control) were added, respectively. The final concentration of MeJA was ~100 μM. The trichomes were harvested after 0 (harvested before treatment) and 6 days for the determination of active ingredient content, and the trichomes were harvested after 0, 0.5, 1, 3, 6, 12, 24, 48, and 72 hours for the determination of gene expression. The surface water was blotted out three times with distilled water. The trichome roots were baked at 45°C to a constant weight (~4–5 days) to determine the change in active ingredient content, and the hair roots were snap-frozen in liquid nitrogen and stored at −80°C for RNA extraction. The experiment included three biological replicates.

### Genome-wide identification of *SmJAZ* genes

Hidden Markov model (HMM) profiles of TIFY (PF06200) and Jas (PF09425) domains were downloaded from Pfam (http://xfam.org/), and used to extract full-length *SmJAZ* candidates from the *S. miltiorrhiza* genome by the HMM algorithm (HMMER) [70], with an E-value <1e^−6^. The Pfam database and the SMART database (http://smart.embl-heidelberg.de/) were used for further analysis to determine if any of the sequences obtained from screening contained the TIFY and Jas domain.

### Phylogenetic analyses

MEGA X was utilized to create phylogenetic trees and multiple sequence alignments [71], using maximum likelihood methods with a bootstrap test (*n* = 500 replications). The gene accession numbers used to build the evolutionary tree are presented in Supplementary Data Table S1, and the gene sequences were obtained from NCBI (https://www.ncbi.nlm.nih.gov/).

Exon–intron distribution prediction was performed using Splign on NCBI (https://www.ncbi.nlm.nih.gov/sutils/splign/splign.cgi), and GSDS 2.0 (http://gsds.cbi.pku.edu.cn/) and Inkscape v0.92 for mapping.

Analysis of conserved structural domains was performed using MEME (http://meme-suite.org/doc/meme-format.html).

Gene promoters were analyzed using PlantCARE (http://bioinformatics.psb.ugent.be/webtools/plantcare/html/).

### qRT-PCR

Total RNA extraction and qRT–PCR were performed using previously established procedures [47]. Primer Premier v5.0 software was used to create gene-specific primers (Supplementary Data Table S3) to analyze the expression of pertinent genes. The *S.* miltiorrhiza actin gene (*SmACT*) served as an internal control and was normalized to the control samples [72]. The relative expression levels of the genes were calculated using the 2^–ΔΔt^ technique [73]. Three separate biological replicates and three separate technical replicates were used to analyze each sample.

### Metabolite analyses

Metabolite analyses followed previously described methods with a Waters 1525 HPLC [47]. The injection volume was 10 μl and column temperature was 30°C. The mobile phase consisted of 0.02% (v/v) phosphoric acid in water (A) and acetonitrile (B), and the flow rate was 1 ml min^−1^. The gradient program was as follows: 0–10 minutes, 5–20% B; 10–20 minutes, 25% B; 20–25 minutes, 20% B; 25–40 minutes, 30% B; 40–45 minutes, 45% B; 45–50 minutes, 50% B; 50–58 minutes, 58% B; 58–67 minutes, 50% B; 67–70 minutes, 60% B; 70–80 minutes, 65% B; 80–85 minutes, 100% B; 85–95 minutes, 5% B. The detection wavelengths for salvianolic acids and tanshinones were 288 and 270 nm, respectively.

### Plant expression vector construction

PCR was used to amplify the entire ORF of the SmJAZs using gene-specific primers (Supplementary Data Table S3). After cloning into the pDONR207 entry vector, the fragment was then cloned into the pK7WG2R destination vector by Gateway technology with the BP and LR Clonase Enzyme Kit (Invitrogen, MA, USA) to create the plant-overexpressing vector.

The amplified fragment was cloned into the pDONR207 entry vector and then cloned into the pK7WG2R destination vector to construct the plant-overexpressing vector by Gateway technology first with the BP Clonase Enzyme Kit and then the LR Clonase Enzyme Kit (Invitrogen, MA, USA).

### Generation of transgenic hairy root lines

Transformation of leaf explants from sterile *S. miltiorrhiza* plants was accomplished using previously published procedures [47]. To detect positive hairy root lines, red fluorescent protein (RFP) expression was evaluated using a fluorescence microscope (Leica DM5000 B, Wetzlar, Germany).

### Positive hairy root lines confirmed by PCR

Genomic DNA of transgenic hairy roots was isolated using the Plant Genomic Extraction Kit (TIANGEN, Beijing, China). In order to verify that the T-DNA had fully integrated into the hairy root line genome, PCR amplification of pK7WG2R-*SmJAZ* plasmids was carried out using the specific primers of *rolB*, *rolC*, and the neomycin phosphotransferase II (*NPT II*) gene of *A. rhizogenes* ATCC15834, as well as the cauliflower mosaic virus (CaMV) 35S promoter and *SmJAZ* sequences (Supplementary Data Table S5).

### Y2H assays

The full-length coding sequences of transcription factor genes (*SmMYC2a*, *SmMYC2b*, *SmMYB36*, *SmMYB39*, *SmPAP1*, *SmTTG1*, *SmERF6*, and *SmWRKY1*) were amplified by PCR with gene-specific primers (Supplementary Data Table S3) and cloned into pDEST-GADT7 by Gateway technology. The *SmJAZ*s were cloned into the pDEST-GBKT7 and pDEST-GADT7 vectors. In this study, analysis of interactions between *SmJAZ*s and between *SmJAZ*s and transcription factors were carried out using yeast strain AH109. As negative controls, the empty pGBKT7 and pGADT7 vectors were co-transformed in parallel. After selection on a synthetic SD-dropout medium lacking leucine and tryptophan (SD/−Leu/−Trp), single transformant colonies were screened for growth on an SD/−Ade/–His/−Leu/−Trp medium. Interactions were observed after incubation for 3 days at 30°C.

### LCI assay

To further validate the interaction between SmJAZs and SmTFs, LCI experiments were performed according to a previously described method [74]. Briefly, the expression vectors nLUC-SmJAZ1/2/3/4/6/8/9 and cLUC-SmMYC2a/SmMYC2b/SmMYB39/SmPAP1 were created. These plasmids were subsequently transformed into GV3101 (pSoup-P19). Finally, the transformed positive *Agrobacterium* was transformed into tobacco (*Nicotiana benthamiana*) leaves. Two to three days after injection, chemiluminescence images showing fluorescence intensity spectra were measured using a plant *in vivo* imaging system (Lumazone Pylon2048B, Princeton, USA).

## Acknowledgements

This work was funded by the National Natural Science Foundation of China (31670295, 31670301, and 81773835).

## Author contributions

P.D.M., J.E.D., and Z.S.L. conceived and designed the research. P.D.M., T.L.P., B.B.L., and M.W. performed the experiments. P.D.M. and B.B.L. analyzed the data and wrote the paper.

## Data availability

The datasets supporting the results of this article are included within the article and its supplementary file.

## Conflict of interest

The authors declare no conflict of interest.

## Supplementary data

Supplementary data is available at *Horticulture Research* online.

## Supplementary Material

Web_Material_uhac166
