# Supplementary material for: Functional pleiotropism, diversity, and redundancy of Salvia miltiorrhiza Bunge JAZ family proteins in jasmonate-induced tanshinone and phenolic acid biosynthesis
Source: Hortic Res. 2022 Jul 25;9:uhac166. doi: 10.1093/hr/uhac166 (PMC9531341; doi:10.1093/hr/uhac166)
Supplement: Web_Material_uhac166 [file web_material_uhac166.zip › Supporting information.pdf]

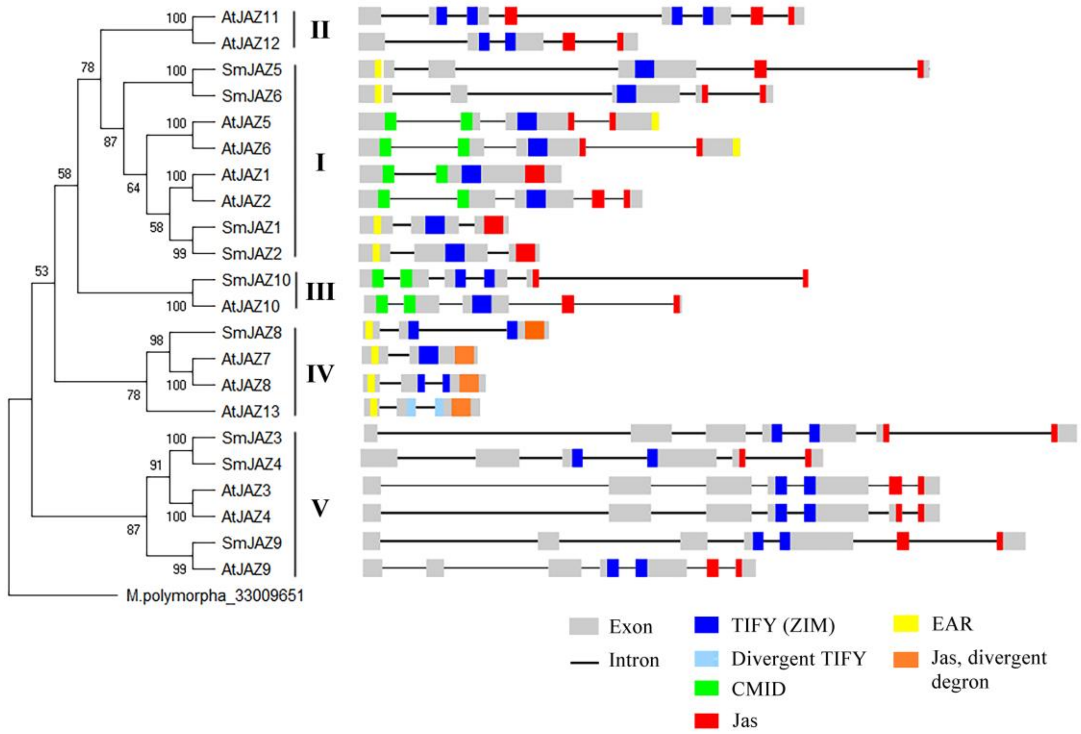

Figure S1. The distribution of exon-intron and conserved domains within SmJAZ family

The various functional elements are shown in different color or shapes.

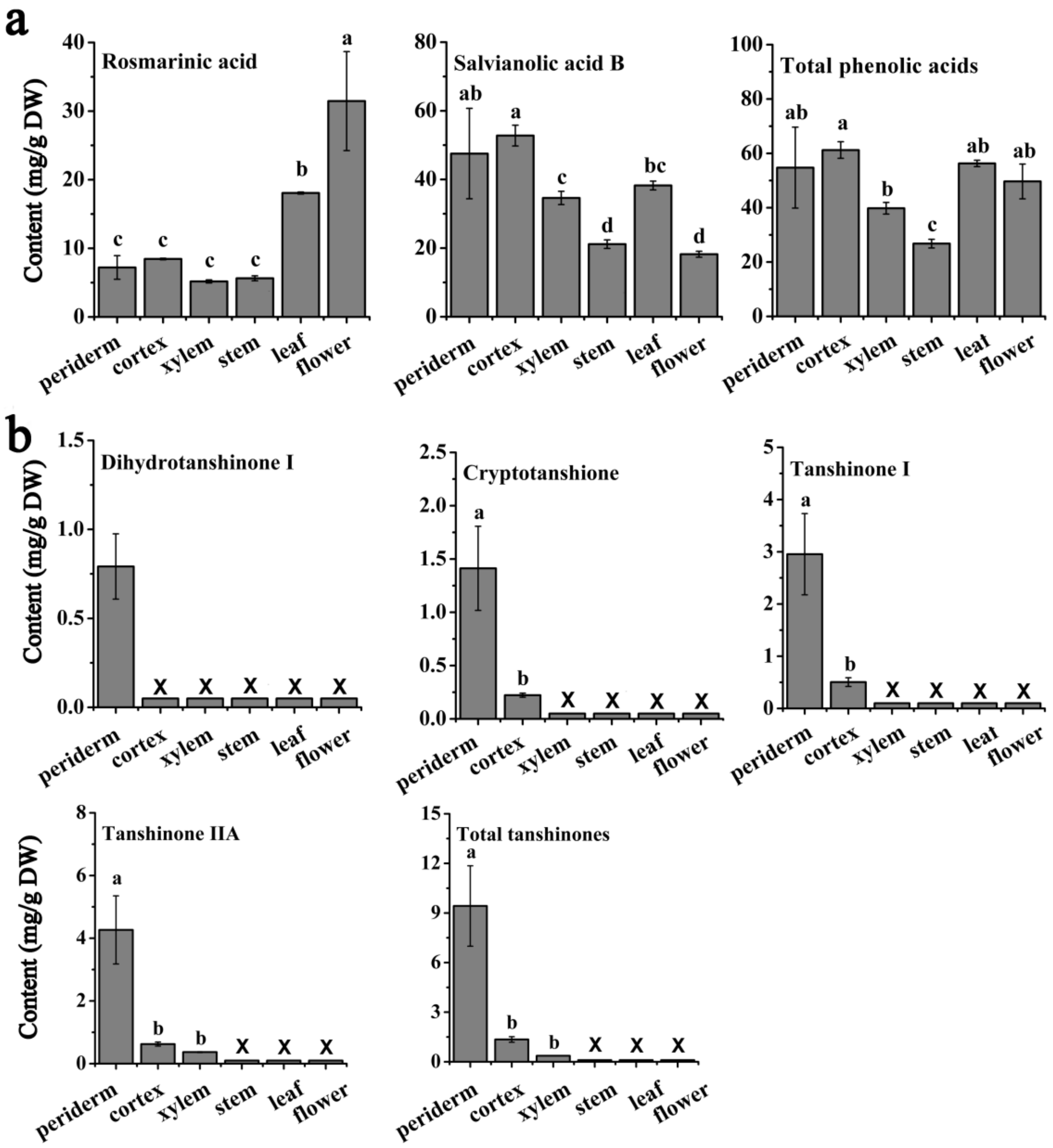

Figure S2. Comparison of salvianolic acids and tanshinones contents from different tissues of *S. miltiorrhiza*

Bars are means ±SD from three independent biological replicates. Different letters indicate the significant differences among the means (P<0.05).

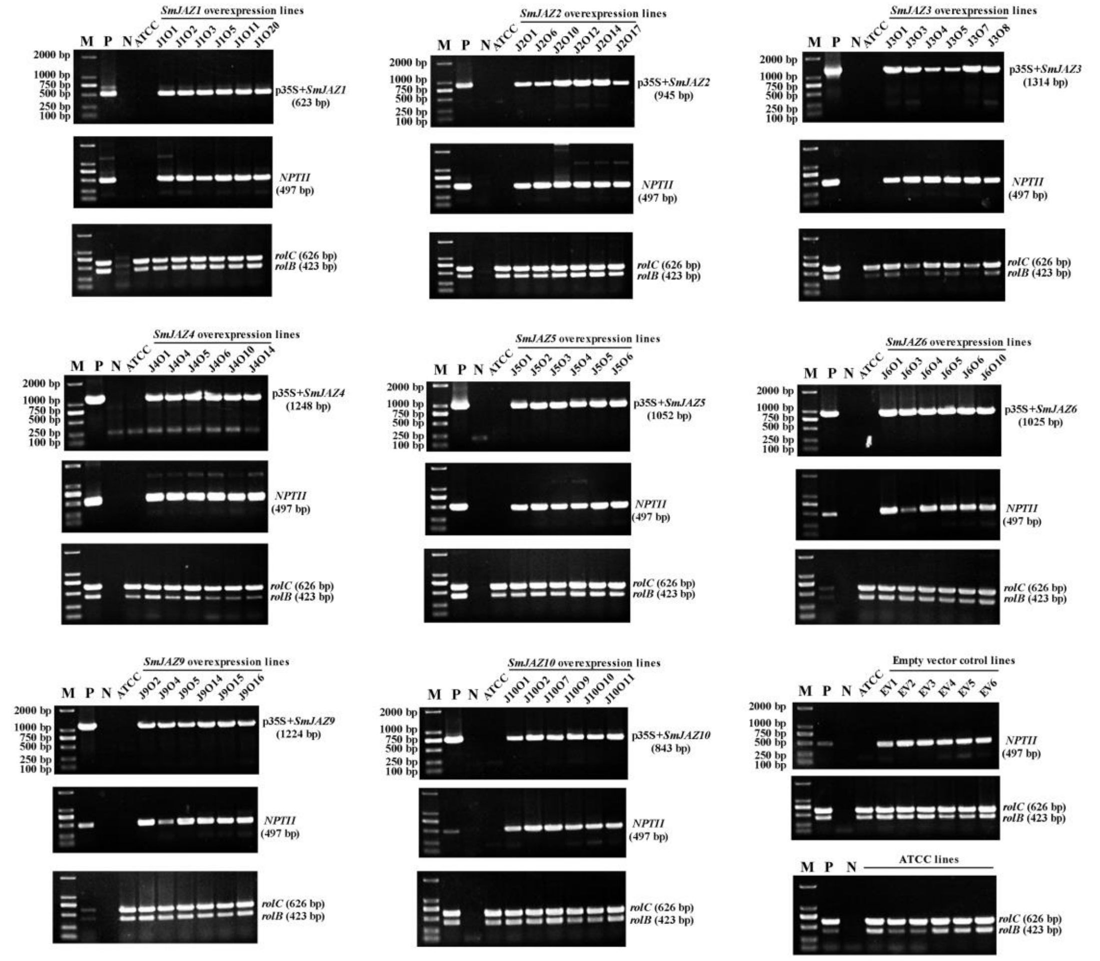

Figure S3. PCR screening of *SmJAZs* overexpressing lines

M DNA Marker; P: ATCC15834 strain which harbours the engineered plasmids (positive control),

N: *S.miltiorrhiza* sterile plantlet (negative control).

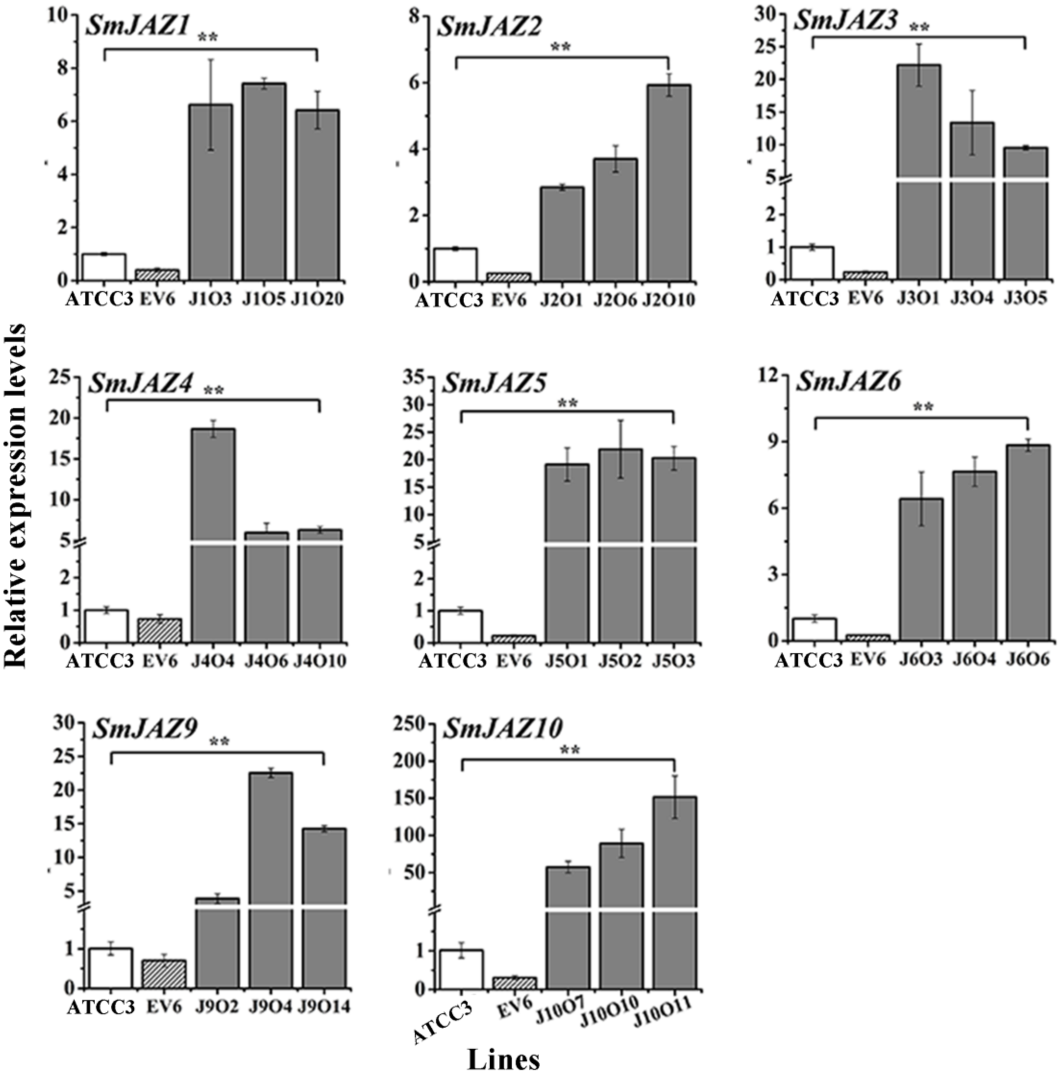

Figure S4. The expression levels of *SmJAZs* in the respective transgenic hairy roots lines

Bars are means±SD from three independent biological replicates. One asterisk (*) indicates a significant difference (0.01<*P*<0.05) and two asterisks (**) indicate a very significant difference (*P*<0.01) between the control and transgenic hairy roots lines. The expression levels of *SmJAZs* in ATCC line were set to 1.

Table S1. The information of sequences used for the phylogenetic tree construction

Lineage	Speices	Gene names	Accession No.	References
Bryophytes	*Marchantia polymorpha*	M.polymorpha_33009651	PTQ32535	(Monte et al., 2018)
	Physcomitrella patens	P.patens_Pp1s15_168V6.1	XP_024399112	(Duan et al., 2013)
		P.patens_Pp1s88_112V6.1	XP_024365014	
		P.patens_Pp1s88_114V6.1	XP_024365843	
		P.patens_Pp1s103_27V6.1	XP_024378255	
		P.patens_Pp1s103_29V6.2	XP_024378251	
		P.patens_Pp1s442_10V6.1	XP_024376421	
		P.patens_Pp1s442_14V6.1	XP_024376075	
Pteridophytes	*Selaginella moellendorffii*	S.moellendorffii_439249	XP_002965453	(Duan et al., 2013)
		S.moellendorffii_406091	XP_002964672	
		S.moellendorffii_447690	XP_002989474	
		S.moellendorffii_415314	XP_002975031	
		S.moellendorffii_407532	XP_024526906	
		S.moellendorffii_418969	EFJ19589	
Monocots	*Oryza sativa*	OsJAZ1	XP_015635689	(Ye et al., 2009)
		OsJAZ2	XP_015646242	
		OsJAZ3	XP_015651049	
		OsJAZ4	XP_015612402	
		OsJAZ5	XP_015634258	
		OsJAZ6	XP_015630632	
		OsJAZ7	XP_015647536	
		OsJAZ8	XP_015610644	
		OsJAZ9	XP_015629221	
		OsJAZ10	XP_015633147	
		OsJAZ11	XP_015633146	
		OsJAZ12	XP_015613048	
		OsJAZ13	XP_015613462	
		OsJAZ14	XP_025876558	
		OsJAZ15	XP_015630229	
	Zea mays	ZmJAZ1	NP_001151145	(Zhang et al., 2015)
		ZmJAZ2	NP_001149525	
		ZmJAZ3	NP_001143811	
		ZmJAZ4	XP_008664988	
		ZmJAZ5	NP_001151261	
		ZmJAZ6	NP_001151346	
		ZmJAZ7	XP_020402586	
		ZmJAZ8	NP_001170674	
		ZmJAZ9	NP_001142202	
		ZmJAZ10	XP_020404895	
		ZmJAZ11	NP_001130163	
		ZmJAZ12	ACF88234	
		ZmJAZ13	NP_001151873	

Lineage	Speices	Gene names	Accession No.	References
		ZmJAZ14	XP_008644871	
		ZmJAZ15	NP_001152572	
		ZmJAZ16	AND66106	
		ZmJAZ17	NP_001141029	
		ZmJAZ18	NP_001182812	
		ZmJAZ19	XP_023156202	
		ZmJAZ20	NP_001308779	
		ZmJAZ21	NP_001150800	
		ZmJAZ22	NP_001150908	
		ZmJAZ23	NP_001313312	
Eudicots	*Arabidopsis thaliana*	AtJAZ1	AT1G19180	(Howe et al., 2018)
		AtJAZ2	AT1G74950	
		AtJAZ3	AT3G17860	
		AtJAZ4	AT1G48500	
		AtJAZ5	AT1G17380	
		AtJAZ6	AT1G72450	
		AtJAZ7	AT2G34600	
		AtJAZ8	AT1G30135	
		AtJAZ9	AT1G70700	
		AtJAZ10	AT5G13220.1	
		AtJAZ11	AT3G43440	
		AtJAZ12	AT5G20900	
		AtJAZ13	AT3G22275	
	Vitis vinifera	VvJAZ1	XM_002284819	(Zhang et al., 2012)
		VvJAZ2	XM_002262714	
		VvJAZ3	XM_003634778	
		VvJAZ4	XM_002272327	
		VvJAZ5	XM_002277733	
		VvJAZ6	XM_002277769	
		VvJAZ7	XM_002277916	
		VvJAZ8	CBI30922	
		VvJAZ9	XM_002277121	
		VvJAZ10	XM_002263220	
		VvJAZ11	XM_002282652	
	Solanum lycopersicum	SlJAZ1	XP_004243696	(Chini et al., 2017)
		SlJAZ2	NP_001234883	
		SlJAZ3	NP_001234223	
		SlJAZ4	XP_004252407	
		SlJAZ5	XP_010318655	
		SlJAZ6	NP_001234373	
		SlJAZ7	NP_001234261	
		SlJAZ8	XP_010322626	
		SlJAZ9	XP_004244920	

Lineage	Speices	Gene names	Accession No.	References
		SlJAZ10	XP_004244919	
		SlJAZ11	XP_004244921	
		SlJAZ12	XP_025884767	

Table S2. Correlation analyses between the expression levels of *SmJAZs* and active ingredient in different organs from *S. miltiorrhiza*

Gene	Compounds							
	RA	Sal B	TPA	DT I	CT	TA I	TA IIA	TTA
SmJAZ1	-0.071	-0.512*	-0.587*	-0.282	-0.329	-0.335	-0.366	-0.344
SmJAZ2	-0.218	-0.010	0.154	-0.105	-0.124	-0.128	-0.154	-0.138
SmJAZ3	-0.195	0.238	0.101	0.724**	0.729**	0.727**	0.741**	0.735**
SmJAZ4	-0.161	0.281	0.171	0.883**	0.882**	0.881**	0.870**	0.878**
SmJAZ5	0.042	-0.334	-0.316	-0.319	-0.373	-0.379	-0.406	-0.386
SmJAZ6	0.106	-0.409	-0.346	-0.421	-0.475*	-0.483*	-0.501*	-0.486*
SmJAZ8	-0.050	-0.272	-0.321	-0.402	-0.449	-0.455	-0.472*	-0.458
SmJAZ9	0.316	-0.360	-0.136	-0.353	-0.418	-0.426	-0.453	-0.431
SmJAZ10	-0.259	-0.332	-0.542*	-0.272	-0.309	-0.314	-0.327	-0.316

RA: Rosmarinic acid; Sal B: Salvianolic acid B; TPA: Total palvianolic acids; DT I: Dihydrotanshinone I; CT: Cryptotanshinone; TA I: Tanshinone I; TA IIA: Tanshinone IIA; TTA: Total tanshinones. One asterisk (*) indicates a significant correlation ($0.01<P<0.05$) and two asterisks (**) indicate a very significant correlation ($P<0.01$).

Table S3. Primers used for qRT-PCR analyses

Gene	Forward (5'–3')	Reverse (5'–3')
SmACT	GGTGCCCTGAGGTCCTGTT	AGGAACCACCGATCCAGACA
SmJAZ1	GGCAGGTCCAACTTCTCGC	TTCTGGTTTCGGCTCACTCG
SmJAZ2	ATGGCTTCCTTTGAGATTGTCG	TCTCCGTAGGAACCCCTTTGA
SmJAZ3	GGTCATTCTCCAACAAGGGC	AACGAACCCCACCTTGCTTC
SmJAZ4	GAGTGGCGATTGCCAAACA	CCGGATAGGTTGTGACCGTG
SmJAZ5	GAGATATCGAATTTCGCCCAA	ATTTTCCAACGCCGTATGC
SmJAZ6	GGTTTTTGATGGCAGGAGGC	CATCAACGGACGATTTCGGA
SmJAZ8	ATGAAGCGCAACTGCAATTT	TGCTGCGTATTGTTCGGACT
SmJAZ9	GTTGAACCCTAAGGATGCTGC	CCCATTCTTCATTGCCTTCTC
SmJAZ10	TTCGGCATGGAGAAAGGATCT	TTATAATCACCCCCACACCGC
SmRAS1	CAGTTTCCGGTGCCCTAAT	TGATGGCGACGAACAAGC
SmCYP98A14	CCTCAACGTCGTCGTTTCCA	AGTCCGCCCAAATCAAATCC
SmCPS1	CCACATCGCCTTCAGGGAAGAAAT	TTTATGCTCGATTTCGCTGCGATCT
SmCYP76AH1	ACGCATCACTTCACCCATCTCA	ATTGCCGACTCATCCACGAT
SmMYC2a	CTAGGAAACGGGGAAGGAAG	TGGACTTGAGCTCGTTGATG
SmMYC2b	GTTGCCAACGGGAATAGAG	GCTTCCTCGGCCTCTTATC
SmMYB36	AAGTGGTCGTTGATCGCCAA	TTTAACCCAATCCAACCCAG
SmMYB39	GCCCTCCCTATCAACAAGAAC	ATCCCAGAAAATCGAATCCAG
SmPAP1	CCGCCGACAAGAAAAAGAT	ATGTCATCGGCGAAGGAAT
SmTTG1	TGCTCAGATTGGCTTGGAATA	GAGTACATGGACATGGGATCA
SmERF6	TCATCCGACCCGAACCGACCCGATT	TAACGCCGCCACGCCATCACAGGAT
SmWRKY1	ACCTACAACGGCCAACACACT	CAATGTCATCCACATCCTCAA

Table S4. Primers used for full-length coding sequences

Gene	Forward (5'–3')	Reverse (5'–3')
SmJAZ1	ATGGTTTCGCCGGAAAAAGTTG	CTAATGGCGCTGAATTTGGAG
SmJAZ2	ATGGCTTCCTTTGAGATTGTCGATT	CTACGGACCCAATCCGAGC
SmJAZ3	ATGGAGAGAGATTTCATGGGTTTGT	TCAATTGGCAGCTGGAACTGG
SmJAZ4	ATGGAGTGGCGATTGCCAAACA	TTAATTCACTGCTGGCACGGAGC
SmJAZ5	ATGTGGAGTACGAAGAACTCATCCCA	CTAATCTTTTTGAGAAGGGAAGT
SmJAZ6	ATGTCTAGTTCAAGAATGGTTTTTGAT	CTAAGACAGTCACAGCATCATAATTT
SmJAZ9	ATGGAGAGAGATTTCATGGGGTTGA	TCAGTCATCCTTGCTGACGGAGACG
SmJAZ10	ATGGTCAAATCTTCATCTCTCG	TTAGTATGGGGACACCGTAATC
SmMYC2a	ATGATTGATTACCGCACGCC	CTATCTAATCTCAGCAACTTTAGAT
SmMYC2b	ATGGGGGTTGTTGGTTGG	TTACCCGAGAGATAACTGATGG
SmMYB36	ATGGCGAGTGATGCATCTCT	TCATTCATCCTCGTCGAGTTC
SmMYB39	ATGGGAAGGTCTCCTTGCTGTG	TCATTTCATCTCCAATCTTCTGTAA
SmPAP1	ATGGGAAGATCCGCTTGCT	TTAAAGAAGCTGCATATACTCGG
SmTTG1	ATGGACAATTCGACCCAGGA	TCAGACTTTGAGCATTTGCATC
SmERF6	ATGATGGCAAATTCTGATGAGG	TCAAGAAGCCGGGTTCGCA
SmWRKY1	ATGGCATCTTCCTCCTGCGT	TCAACTTAAGGTTTCAAATTCGA

Table S5. Primers used for the identification of positive transgenic lines

Gene	Forward (5'–3')	Reverse (5'–3')
rolB	GCTCTTGCAGTGCTAGATTT	GAAGGTGCAAGCTACCTCTC
rolC	CTCCTGACATCAAACTCGTC	TGCTTCGAGTTATGGGTACA
NPTII	ACGTTGTCACTGAAGCGGGAAGG	GGCGATACCGTAAAGCACGAGGAA
p35S+*SmJAZ1*	GACGCACAATCCCACTATCC	CTAATGGCGCTGAATTTGGAG
p35S+*SmJAZ2*	GACGCACAATCCCACTATCC	CTACGGACCCAATCCGAGC
p35S+*SmJAZ3*	GACGCACAATCCCACTATCC	TCAATTGGCAGCTGGAACTGG
p35S+*SmJAZ4*	GACGCACAATCCCACTATCC	TTAATTCACTGCTGGCACGGAGC
p35S+*SmJAZ5*	GACGCACAATCCCACTATCC	CTAATCTTTTTGAGAAGGGAAGT
p35S+*SmJAZ6*	GACGCACAATCCCACTATCC	CTAAGACAGTCACAGCATCATAATTT
p35S+*SmJAZ9*	GACGCACAATCCCACTATCC	TCAGTCATCCTTGCTGACGGAGACG
p35S+*SmJAZ10*	GACGCACAATCCCACTATCC	TTAGTATGGGGACACCGTAATC

Table S6. Cis-elements contained in the promoter region of SmJAZs

Cis-element	Function	Genes
CGTCA-motif	MeJA responsive element	*SmJAZ1/2/5/6* (+/-)[c], *SmJAZ3* (-)[b]
TGACG-motif		*SmJAZ1/2/5/6* (+/-), *SmJAZ3* (+)[a]
ABRE	ABA responsive element	*SmJAZ1/2/4 /5/6/8/10* (+/-), *SmJAZ3* (+)
TGA-element	Aux responsive element	*SmJAZ2* (+/-), *SmJAZ3* (+), *SmJAZ5* (-)
TATC-box	GA responsive element	*SmJAZ4* (-)
ERE	ETH responsive element	*SmJAZ2/5* (-), *SmJAZ4* (+), *SmJAZ8/9* (+/-)
TCA-element	SA responsive element	*SmJAZ3/10* (-), *SmJAZ9* (+)
WUN-motif	Wound responsive element	*SmJAZ3* (+), *SmJAZ4/6* (-)
LTR	Low-temperature responsed element	*SmJAZ1* (+/-), *SmJAZ5* (-), *SmJAZ10* (+)
MBS	Drought responsive element	*SmJAZ2* (-), *SmJAZ5* (+), *SmJAZ9/10* (+/-)
ARE	Anoxic stress responsive element	*SmJAZ1/3/10* (-),*SmJAZ2* (+/-), *SmJAZ5/6* (+)
GC-motif		*SmJAZ8* (+)
TC-rich repeats	Stress responsive element	*SmJAZ3* (+), *SmJAZ4/10* (-)
Box 4	Light responsive element	*SmJAZ1/4/8* (+/-),*SmJAZ2/3/5 /9* (-), *SmJAZ6* (+)
G-box		*SmJAZ1/2/4/9/10* (+/-), *SmJAZ3/5/6/8* (-)
GT1-motif		*SmJAZ1/4* (+), *SmJAZ5/6* (+/-), *SmJAZ8* (-)
I-box		*SmJAZ1/3* (+)
LAMP-element		*SmJAZ1/4/9* (+)
Sp1		*SmJAZ1/4* (+), *SmJAZ3* (-)
TCT-motif		*SmJAZ1/8* (-), *SmJAZ3/6* (+)
ACE		*SmJAZ2/9* (-)
AE-box		*SmJAZ2/3* (-), *SmJAZ4* (+/-)
TCCC-motif		*SmJAZ2* (+/-), *SmJAZ6/9* (+)
AT1-motif		*SmJAZ3* (-)
GATA-motif		*SmJAZ4/10* (-), *SmJAZ8* (+)
MRE		*SmJAZ4/10* (-), *SmJAZ9* (+)
ATCT-motif		*SmJAZ6/8* (-)
CAT-box	Meristem expression-related element	*SmJAZ2/4* (+), *SmJAZ9* (-)
MSA-like	Cell cycle regulated element	*SmJAZ2* (-)
O2-site	Zein metabolism regulation	*SmJAZ2* (-), *SmJAZ5* (+)
circadian	circadian control	*SmJAZ4* (-)
GCN4-motif	endosperm expression-	*SmJAZ6* (+/-), *SmJAZ8* (+),

	related element	*SmJAZ10* (-)

a, “+” represents that the element is located in the DNA sense strand; b, “-” represents that the element is located in the DNA anti-sense strand; c, “+/-” represents that the element is located in both the DNA sense and anti-sense strand.
